# Specific algorithm method of scoring the Clock Drawing Test applied in cognitively normal elderly

**DOI:** 10.1590/1980-57642015DN92000007

**Published:** 2015

**Authors:** Liana Chaves Mendes-Santos, Daniel Mograbi, Bárbara Spenciere, Helenice Charchat-Fichman

**Affiliations:** 1 PhD, Department of Psychology, Pontifical Catholic University of Rio de Janeiro RJ, Brazil.; 2 Department of Psychology, Institute of Psychiatry, King’s College London, UK.; 3 BsC, Department of Psychology, Pontifical Catholic University of Rio de Janeiro RJ, Brazil.

**Keywords:** Clock Drawing Test, inter-rater reliability, elderly, neuropsychology

## Abstract

The Clock Drawing Test (CDT) is an inexpensive, fast and easily administered measure of cognitive function, especially in the elderly. This instrument is a popular clinical tool widely used in screening for cognitive disorders and dementia. The CDT can be applied in different ways and scoring procedures also vary.

**Objective:**

The aims of this study were to analyze the performance of elderly on the CDT and evaluate inter-rater reliability of the CDT scored by using a specific algorithm method adapted from Sunderland et al. (1989).

**Methods:**

We analyzed the CDT of 100 cognitively normal elderly aged 60 years or older. The CDT ("free-drawn") and Mini-Mental State Examination (MMSE) were administered to all participants. Six independent examiners scored the CDT of 30 participants to evaluate inter-rater reliability.

**Results and Conclusion:**

A score of 5 on the proposed algorithm ("Numbers in reverse order or concentrated"), equivalent to 5 points on the original Sunderland scale, was the most frequent (53.5%). The CDT specific algorithm method used had high inter-rater reliability (p<0.01), and mean score ranged from 5.06 to 5.96. The high frequency of an overall score of 5 points may suggest the need to create more nuanced evaluation criteria, which are sensitive to differences in levels of impairment in visuoconstructive and executive abilities during aging.

## Abstract (pt)

O Teste do Desenho do Relógio (TDR) é uma barata e rápida medida de função cognitiva, de fácil aplicação, especialmente em idosos. Este instrumento é uma ferramenta clínica muito conhecida, amplamente utilizada no rastreamento de transtornos cognitivos e demência. O TDR pode ser aplicado de diferentes formas e a sua pontuação também varia.

**Objetivo:**

Os objetivos deste estudo foram analisar o desempenho dos idosos no TDR e avaliar a confiabilidade inter-examinadores do TDR pontuado por um método com algoritmo específico, adaptado a partir dos critérios estabelecidos por Sunderland et al. (1989).

**Métodos:**

Analisamos o TDR de 100 idosos cognitivamente saudáveis com 60 anos de idade ou mais. O TDR ("desenho livre") e o Mini-Exame do Estado Mental (MEEM) foram administrados em todos os participantes. Seis avaliadores independentes pontuaram 30 TDR para avaliar a confiabilidade inter-examinadores.

**Resultados e Conclusão:**

A pontuação 5 do algoritmo proposto ("Os números em ordem inversa ou concentrados") equivalente a 5 pontos na escala original de Sunderland foi a mais frequente (53,5%). O método com algoritmo específico do TDR utilizado teve alta confiabilidade entre avaliadores (p<0,01), e a média da pontuação variou entre 5,06 e 5,96. A alta frequência de 5 pontos na pontuação geral pode sugerir a necessidade da elaboração de critérios de avaliação mais sutis, que sejam sensíveis às diferenças entre indícios de comprometimento nas habilidades visuoconstrutivas e executivas durante o envelhecimento.

## INTRODUCTION

The Clock Drawing Test (CDT) is a simple and ecological neuropsychological instrument that covers a wide range of cognitive functions, including selective and sustained attention, auditory comprehension, verbal working memory, numerical knowledge, visual memory and reconstruction, visuospatial abilities, on-demand motor execution (praxis) and executive function.^1^ Some studies have demonstrated the robust psychometric properties of the CDT.^2-4^

The CDT has been used as a cognitive screening tool, particularly in the elderly population, to differentiate cognitively normal individuals from individuals with cognitive impairment and dementia.^5-7^ This test has the capacity to evaluate multi-domain impairments that may go undetected by other cognitive screening instruments, such as the Mini-Mental State Examination (MMSE).^2,8^ The relative independence of verbal abilities^9,10^ makes it especially useful in patients presenting marked verbal impairment or aphasia. In addition, the CDT has also shown strong associations with other cognitive measures, such as the Cambridge Cognitive Examination (CAMCOG),^6,11,12^ justifying the inclusion of the CDT in several neuropsychological cognitive screening batteries.^1,10,12^

Although there is great interest in CDT as a screening instrument, a wide variety of CDTs have been developed, each relying on different systems of administration and quantitative or qualitative error scoring, with no consensus on which system produces the most valid results.^3,5,13^ The currently used methods are Shulman et al.,^14^ Sunderland et al.^10^ and Mendez et al.^1-3,15^ These different applications and systems of scoring are somewhat similar and highly correlated in some aspects, but their diagnostic accuracy, and the cognitive processes involved in their performance are different.^16^

CDT performance is associated with several brain regions, including the bilateral parietal lobes, right and left posterior and middle temporal lobes, right middle frontal gyrus, and the right occipital lobe.^16,17^ These areas can also be associated with a broad spectrum of pathologies. A number of studies have shown the potential of the CDT for investigating cognitive performance in patients with schizophrenia, Alzheimer's disease, Parkinson's disease, depression and other disorders.^9,18,19^

Previous studies have investigated the test-retest reliability,^1,9^ and determined inter-rater reliability, of the CDT.^6,10,20-24^ These studies compared the different application systems and showed that the systems were well correlated, took little time and had high inter-rater reliability. On the other hand, CDT reliability has rarely been assessed in population-based studies, particularly in developing countries. Three studies determining inter-rater reliabilities of the CDT in elderly in Brazil were found: one scored the tests with Shulman's method,^20^ while the others compared the accuracy of scales (Mendez, Shulman and Sunderland;^6^ Sunderland, Shulman, Manos & Wu and Wolf-Klein^24^) and determined the inter-rater reliability of CDT performance. These investigations showed good inter-rater reliabilities.

One of the most used methods of CDT scoring is Sunderland et al.^10^ This method of scoring is well established in the literature^10,25-27^ and widely used in Brazil, being part of cognitive screening batteries for the elderly.^28,29^

With the aim of providing a more detailed, specific and quantitative analysis of the different aspects of CDT assessment, the present study proposed an algorithm method for scoring the CDT adapted from Sunderland et al.^10^ To this end, the performance of 100 elderly was analyzed using the new algorithm, and its inter-rater reliability was evaluated.

## METHODS

**Participants.** The sample was part of a larger study involving 350 elderly from community centers, known as "Casas de Convivência", belonging to the Rio de Janeiro municipal administration. One hundred elderly took part in this study (93 females and seven males). The inclusion criteria were:

[1] to be literate (a person who can read and write; mean=9.8 years of education, SD=4.2),
[2] to be aged 60 years or older (mean age=72.6 years old, SD=5.9), and
[3] to be cognitively healthy (MMSE mean score=25.3, SD=3.4).

Cut-off scores for the MMSE were defined according to educational level. MMSE scores range from 0 to 30, with higher scores indicating better cognitive function; the cut-off for cognitive impairment was 18 in individuals with fewer than four years of formal education and 24 for participants with more than four years of education.^8,30^ Exclusion criteria were: to be visually and/or hearing impaired or have uncorrected deficits, presence of endocrine and metabolic abnormalities, neurological and psychiatric disorders, or difficulty executing hand movements due to rheumatic diseases.

Before entry to the study all participants received an explanation on the objectives of the research, and signed an informed consent form. The Research Ethics Committee of the State University of Rio de Janeiro approved this study.

**Materials and procedures.** Subjects were first submitted to a standardized questionnaire, which collected data on sociodemographic variables (i.e., gender, age and education), on subjective memory impairment (i.e., "Do you feel like your memory has gotten worse?"), and on comorbidities. All participants then completed the same protocol of cognitive screening tests. The tests were applied in the following sequence (based on Nitrini et al.^31^):

[1] MMSE;^8,30^
[2] Memory Test Figures;^31^
[3] Verbal Fluency – Animals;^9,32^
[4] CDT (described below);
[5] The Lawton Instrumental Activities of Daily Living^33,34^ (for further details see Charchat-Fichman et al.^35^). Besides the cognitive and functional evaluations, participants completed the Geriatric Depression Scale (GDS).^36^

The CDT was applied to all participants in the spontaneous modality that uses a pencil and blank sheet of paper. The patients were asked to draw a clock without a model. Trained examiners issued a standardized instruction: "Draw a clock, put in all the numbers, and set the hands to 2 hours and 45 minutes." There was no time limit.

**Figure 1:**
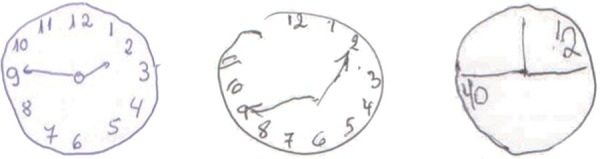
Examples of CDT score in accordance with the specific algorithm method based on Sunderland et al.^10^: 9, 5 and 2 (right to left), respectively.

Table 1 shows the original CDT scoring scale by Sunderland et al.,^10^ which forms the basis of the new algorithm (Table 2). Both Tables 1 and 2 present the correspondence of higher scores indicating better performance. Examples of the CDT scoring scale by Sunderland et al.^10^ are given in Figure 1. According to the new algorithm (Table 2), the examiner must first mark with an "X" all the items present in the clock drawing. The list of items has increasing complexity.

**Table 1:** The original Sunderland method for scoring the CDT.^10^

10-6	Drawing of clock face with circle and numbers is generally intact.
10	Hands are in correct position.
9	Slight errors in placement of hands.
8	More noticeable errors in placement of hour and minute hands.
7	Placement of hands is significantly off course.
6	Inappropriate use of clock hands (i.e., use of digital display or circling of numbers despite repeated instructions).
5-1	Drawing of a clock face with circle and numbers is not intact.
5	Crowding of numbers at one end of the clock or reversal of numbers. Hands may still be present in some fashion.
4	Further distortion of number sequence. Integrity of clock face is now gone (i.e., numbers missing or placed at outside of the boundaries of the clock face).
3	Numbers and clock face no longer obviously connected in drawing. Hands are not present.
2	Drawing reveals some evidence of instructions being received but only a vague representation of a clock.
1	Either no attempt or an uninterpretable effort is made.

**Table 2:** New algorithm method for CDT scoring based on the original criteria of Sunderland et al.^10^

You should mark with an “X” all the items present in the clock drawn				
(a)	Presence of circle.		(j)	Presence of hour hand.
(b)	Presence of 12 numbers.		(k)	Presence of minute hand.
(c)	Numbers entered in the internal limit of the clock.		(l)	Minute hand proportionally larger than the hour hand.
(d)	Number in the correct ascending order.		(m)	One of the hands between 2 and 3.
(e)	Numbers in correct spatial position.		(n)	One of the hands on exactly 9.
(f)	Can you draw a straight vertical line between 12 and 6.		(o)	Wrong use of hands (digital or circling the numbers).
(g)	Can you draw a straight horizontal line between 3 and 9.		(p)	Some evidence of having understood that it is a clock.
(h)	Numbers not concentrated in one part of the clock.		(q)	Did not try or did not represent a clock.
(i)	Presence of two pointers.			
**Follow the algorithm for the score, but consider these three points initially**				
1. If the item “o” is checked, the score is 6 points.				
2. If the item “p” is checked, the score is 2 points.				
3. If the item “q” is checked, the score is 1 point.				
**The score will be 10-6 if the clock and the numbers are drawn correctly**				
10	Correct time (no “X” in the items: “o”, “p”, “q”).			
9	Very mild disorder of hands (absence of “X” in at least one item: “l”, “m” or “n”).			
8	Mild disorder of hands (absence of “X” in at least 2 items: “l”, “m”, “n”).			
7	Severe disorder of hands (absence of “X” in the items: “l”, “m”, “n”).			
6	Wrong use of hands (presence of “X” in item “o”).			
**The score will be 5-1 if the drawing of the clock and the numbers are incorrect**				
5	Numbers in reverse order or concentrated (no “X” in the items: “d” or “h”).			
4	Numbers missing and located outside the boundary of the clock (no “X” in items: “b” and “c”).			
3	Absence of hands (no “X” in the items: “i”, “j”, “k”).			
2	Only some evidence of having understood that it is a clock (presence of “X” in item p).			
1	Not tried or did not represent a clock (presence of “X” item in q).			

Inter-rater reliability was assessed by comparing CDT scores from six independent examiners, who each scored the same 30 clocks from subjects sampled randomly.

## RESULTS

A summary of the participants' sociodemographic characteristics, performance on cognitive screening tests, as well as cognitive function and depression scales is given in Table 3. Table 4 shows performance on the CDT.

**Table 3:** Participants’ sociodemographic characteristics, and performance on cognitive screening tests, as well as cognitive function and depression scales.

Sociodemographic characteristics		Mean	SD*	Minimum value	Maximum value
Age		72.6	5.9	60	84
Years of education		9.8	4.2	3	24
Instruments and scales	MMSE (Memory Figures Test)	25.2	3.3	18	30
	• Incidental Memory	25.4	1.1	2	8
	• Immediate Memory 1	7.9	1.3	4	10
	• Immediate Memory 2	8.6	1.1	5	10
	• 5 Minutes - Delayed Memory	7.7	1.5	4	10
	• Recognition	9.9	0.3	8	10
Verbal Fluency		15	4.8	5	27
Lawton’s Scale		20.1	1.4	18	21
GDS		1.9	2.1	0	8

* SD: standard deviation.

**Table 4:** Participants’ performance on CDT: mean, median, standard deviation, minimum and maximum score.

N	Mean	Median	Standard deviation	Minimum score	Maximum score
100	5.22	5	2.02	2	10

**Figure 2:**
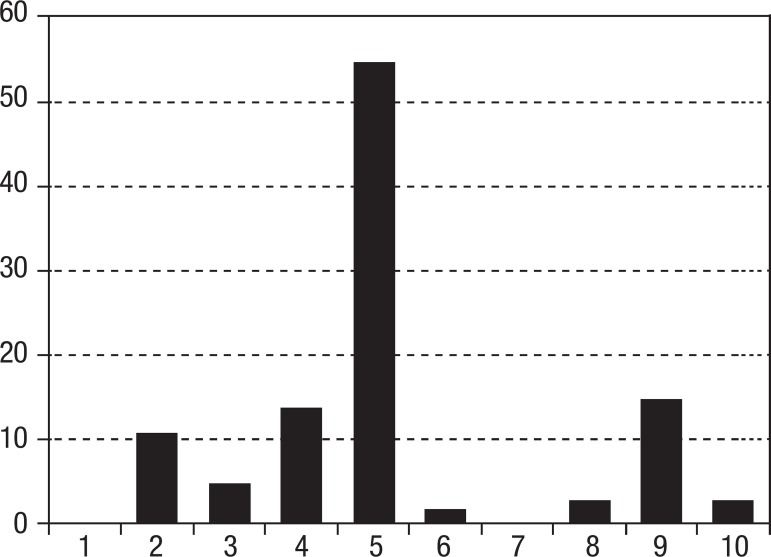
Histogram showing the frequency of CDT scores according to the scoring system developed by Sunderland et al.^10^.

According to the histogram shown in Figure 2, regarding the performance of the elderly on the CDT, the frequency of score "5" was 53.5%, and scores "1" and "7" were not present in the current sample.

Pearson's correlation was used to evaluate the relationship between schooling, age and MMSE with CDT scores. No significant correlation was found between schooling and CDT (r=0.014, p>0.05) or age and CDT (r=0.04, p>0.05), but a significant positive correlation was found between MMSE and CDT (r=0.22, p<0.05).

On the other hand, the investigation of inter-rater reliability of the CDT, scored by criteria based on Sunderland et al.,^10^ showed that the mean ranged from 5.06 to 5.96 (Table 5).

**Table 5:** Mean and SD of CDT scores rated by the six examiners.

Examiners	Mean	SD
1	5.06	2.24
2	5.66	2.57
3	5.96	2.74
4	5.73	2.55
5	5.23	1.95
6	5.6	2.71

SD: standard deviation.

Pearson's correlation analysis was performed between the scores found by the six independent raters for 30 tests. A significant positive correlation was found between the examiners (p<0.01): 1 and 2 (r=0.79), 1 and 3 (r=0.7); 1 and 4 (r=0.75); 1 and 5 (r=0.84), 1 and 6 (r=0.71), 2 and 3 (r=0.87), 2 and 4 (r=0.79), 2 and 5 (r= 0.79), 2 and 6 (r=0.79), 3 and 4 (r=0.79), 3 and 5 (r=0.69), 3 and 6 (r=0.8), 4 and 5 (r=0.79), 4 and 6 (r=0.88) and 5 and 6 (r=0.74).

The agreement between raters was high, consistently remaining statistically significantly above expected chance agreement (in all cases, p<0.001). The combined kappa for all six examiners was 0.60, with pairwise analyses between evaluators indicating an average level of agreement of 90.2% and an average weighted kappa of 0.69.

## DISCUSSION

The current study analysed the performance of a cognitively normal elderly community sample on the CDT using a specific algorithm score method based on the Sunderland et al.^10^ system. The mean score of participants was 5.22, and the standard deviation 2.02. The score 5 ("Numbers in reverse order or concentrated") was observed in 53.5% of clock drawings.

In general, studies with the CDT compare the performance of patients and controls in different applications and scoring systems^2,25,37^ or verify the clinical validity of the test,^21,23,38^ or investigate the translation and adaptation of the CDT model for a particular population.^39,40^ There are few studies in community-dwelling samples or cognitively normal elderly.^22,41-43^

Five Brazilian studies using Sunderland's scoring method found higher scores than the present study (5.22, and standard deviation 2.02). Two of these studies did not mention CDT scores,^6,24^ while the other results were: 9.7 (±1.07),^41^ 7.8 (±2.2),^28^ and 9.1 (±1.8).^11^ However, comparison of the current findings with results of these studies is hampered because of a number of differences in study design. The most important difference was related to the intrinsic characteristics of the sample. The cited studies used small clinical samples recruited in hospital settings, in contrast to the present study which used a large sample of normal elderly from community centers with heterogeneous age and educational levels.^6,11,24,28,31,41^ The objectives of the studies also varied. Some compared different methods of CDT scoring,^24,41^ others compared the instrument with other tests and finally there was a study that evaluated the profile of the elderly subjects on the CDT^28^ based on a selected group of normal elderly as a control group compared to Alzheimer's disease patients.

Studies in the international literature that used the same method as Sunderland to score the CDT found the following results: 7.5 (±1.9),^25^ 8.4 (±1.6),^27^ 8.7 (±1.1),^10^ and 8.9 (±1.4).^26^ Similar to the Brazilian studies, all of these found higher scores for normal elderly individuals^10,26,27^ than in the present study, except Kirby et al.^25^ who found lower scores compared to the other international studies. Some studies failed to mention all important information, for example, the educational level^10,26^ or did not use formal cognitive testing for normal controls^10^ (including the MMSE^10,27^) while another did not separate the clinical group when describing the sample characteristics,^27^ hindering comparisons among the studies. The aim of the present study differs from the main objective of the previous studies in that its aim was to evaluate the performance of the elderly with and without cognitive impairment.^10,25-27^

An important outcome regarding the performance of the elderly is the high percentage (53.5%) of the sample with scores of "5". The criterion for a score of "5" in Sunderland's original method is "Crowding of numbers at one end of the clock or reversal of numbers. Clock Hands may still be present in some fashion" and in the new algorithm denoted: "Numbers in reverse order or concentrated". The lower mean scores on the CDT compared to other studies, and the high frequency of elderly that scored at this level could be explained by the fact that strict correction was used to score the CDT in this study. Sunderland's method in its original version had a more subjective approach, for example, very high CDT scores, even with numbers slightly concentrated, could be found in Sunderland et al.^10^ (Figure 1, p. 727). According to Sunderland's method, item 5 should be scored only when there is a drastic concentration, and in the present research this item included people with slight and severe difficulty in planning. Thus, when strict criteria are used, different results are obtained compared to the literature.

In this sense, it would be necessary to develop more specific scoring criteria that may be sensitive to planning strategy and visual-constructive execution of the CDT, and which could better differentiate specifically those elderly with possible executive dysfunction. Other methods of scoring the CDT, including semi-quantitative and qualitative scoring systems, attempt to discriminate the level of executive planning in clock drawings,^42,44,45^ and emphasize the evaluation of executive components in CDT.^42-44^ For example, Royall et al.^45^ developed the Executive Clock Drawing Task (CLOX) in order to discriminate these components and allow a more specific analysis of how the executive functions can be tested in the CDT.

No significant correlation was found between education or aging and CDT scores. The relationship between education, aging and CDT performance is controversial in the literature.^22,24,38,41,43^ This finding may also be related to the existence of various application methods and different scoring scales. For example, Brodaty and Moore found a correlation of CDT score with years of education for the Shulman and Sunderland, but not for the Wolf-Klein scoring system.^2^ Sunderland et al.^10^ did not report the educational level of control subjects in the original study.

On the other hand, a significant positive correlation was found between the CDT and MMSE, confirming previous findings.^6,7,15^ A high correlation has been found for the scales of Shulman,^14^ Mendez^1^ and the CLOX scale.^45^ The association between MMSE score and several CDTs was also high in the study by Schramm et al.^7^

These various systems of application and scoring are an obstacle to establishing direct comparisons and drawing conclusions. The different forms of application include differences in the clock time requested (2:45, 11:10, 8:05) and presence of drawing assistance (e.g. some have a pre-drawn circle). In addition, the various scoring systems include: 10 hierarchical patterns (0-10), scale based on errors each scored 0/1 (0-20), clock divided into eighths, points given for numbers and hands in right place (0-10) and others.^3,14,37,43^

In this study, an algorithm with more specific scores based on Sunderland et al.^10^ criteria was devised to increase inter-rater reliability. The examination of the inter-rater reliability showed that the criteria developed for the present study were reliable and a significant positive correlation was found between the six independent examiners. These results are similar to those found in previous studies, also indicating high inter-rater reliability of CDT scores.^10,21-23^ Again, the various ways of presenting the test and the different principles involved in scoring, make comparisons difficult. Another aspect that hampers comparisons is the use of several different study designs. Some studies examined inter-rater reliabilities of the CDT scored by one scoring system in cognitively normal elderly^20^ or in differentiating between cognitively normal and individuals with different types of pathologies,^2^ while others examined inter-rater reliability using different scoring systems among cognitively normal elderly^22,37^ or cognitively normal and individuals with different types of diseases.^21^ Two other studies that evaluated the inter-rater reliability using various score systems, including the method of Sunderland et al.,^10^ compared subjects with and without pathologies (fibromyalgia and mild cognitive impairment, MCI)^37,46^ and showed good inter-rater reliability.

The idea of systematic scoring of the CDT has focused on the development and standardization of simple and easy-to-interpret scoring methods.^21,22^ There are two general CDT scoring approaches, including qualitative and quantitative approaches. The Sunderland et al.^10^ is a semi-quantitative scoring system that focuses on scoring the whole clock.^37^ Other quantitative scoring systems focus on different aspects of the clocks (such as clock face, numbers or hands) and score them separately (i.e., the Clock Drawing Interpretation Scale by Mendez et al.^1^ and Rouleau et al.^12^). Furthermore, the scoring systems differ regarding scoring procedures.

One limitation of this study is the non-stratification of participants by age for comparison. Perhaps the advanced age of some participants may have influenced the low average scores. Another question to be considered centers on the intrinsic characteristics of the sample and on the volunteers that participated in the activities of the Casas de Convivência. For example, the sample comprises mostly women (93%), with few health conditions. However, considering this is a convenience sample, it was not possible to limit recruitment on the basis of personal characteristics In addition, other Brazilian studies also feature a higher percentage of women,^11,20,24,41^ making it unlikely that this represents a major bias in results. These subjects were normal elderly (criterion for inclusion in the sample was to score above the cut-off point on the MMSE), but some older adults with MCI might have been included in the sample; a number of conditions associated with aging could be present, and some comorbidities not directly related with cognition may have influenced the results. Another limitation to be considered is associated with the method of sample selection. To adequately address selection bias, a randomized sample would have been better than the convenience sample used in the present study. Moreover, other limitations were the absence of other measures of executive functions to compare with the CDT and no functional literacy examination.

The present findings represent an important contribution to the discussion on which CDT administration and scoring system produces the most valid results. The results confirmed the consistency of the scoring criteria of Sunderland et al.^10^. Furthermore, the findings contribute to the discussion about the lack of consensus on the different scoring criteria developed for the CDT and on which would produce more valid results. On the other hand, they may further suggest the need for creating more subtle evaluation criteria, which are sensitive to the differences between impairment in visuoconstructive and executive abilities during aging.

Future research should replicate these findings in elderly with higher and lower formal education to compare the impact of educational level on the CDT. Additional studies could explore more qualitative aspects of the CDT, including strategies implemented, as well as comparing it to other scoring criteria, and clinical validation in the case of Alzheimer's disease, MCI and depression.
